# Agreement of self- and physician-collected samples for detection of high-risk human papillomavirus infections in women attending a colposcopy clinic in Thailand

**DOI:** 10.1186/s13104-018-3241-9

**Published:** 2018-02-20

**Authors:** Natacha Phoolcharoen, Nuttavut Kantathavorn, Wasanai Krisorakun, Thaniya Sricharunrat, Narongchai Teerayathanakul, Chantanee Taepisitpong, Gaidganok Sornsamdang, Waraphorn Krongthong, Siriporn Saeloo

**Affiliations:** 1 Chulabhorn Hospital, HRH Princess Chulabhorn College of Medical Science, Chulabhorn Royal Academy, Bangkok, Thailand; 2 Data Management Unit, HRH Princess Chulabhorn College of Medical Science, Chulabhorn Royal Academy, Bangkok, Thailand; 3 Department of Obstetrics and Gynecology, Faculty of Medicine, Chulalongkorn University, 1873 Rama IV Road, Pathum Wan, Bangkok, 10330 Thailand

**Keywords:** Self-sampling, HPV testing, Cervical cancer screening, Colposcopy, Thailand

## Abstract

**Objective:**

To study the concordance between vaginal self- and endocervical physician-collected high-risk (hr) HPV testing in Thai women who attended a colposcopy clinic. Vaginal samples were obtained by self-sampling with a dry brush before endocervical samples were obtained by physicians. Both specimens were analyzed for hrHPV by Cobas4800 HPV test.

**Results:**

Of the 247 pairs of samples, overall hrHPV prevalence from self- and physician-collected samples was 41.3 and 36.0%, respectively. The overall agreement between the methods was 74.5% with κ 0.46 (*P* < 0.001). Our study revealed moderate agreement between self- and physician-collected methods for hrHPV testing.

## Introduction

Worldwide trends in incidence and mortality rate of cervical cancer have decreased as a result of effective organized screening programs, however, cervical cancer remains an important health problem in less-developed countries. In Thailand, it is the second most common cancer in women, with an age-standardized incidence rate of 17.8 per 100,000 [1, 2].

The Ministry of Public Health in Thailand launched a national screening program for women aged 30–60 years since 2002 but the coverage rate was 46–67%, which was lower than the target of 75% [3–5]. The main reasons for avoiding cervical cancer screening in Thai women were embarrassment and fear of pain or fear of vaginal examination [6, 7].

Human papillomavirus (HPV) testing was approved for primary screening because of its satisfactory sensitivity for detecting high-grade precancerous cervical lesions [8–10]. Self-sampling HPV testing has been increasingly adopted for cervical cancer screening [11, 12]. Many studies have shown the advantage of self-sampling in increasing screening attendance and coverage [13–18]. Most studies have revealed high agreement between HPV screening results from self- and physician-collected specimens [19–25] and positive acceptability and preferences among women [26, 27].

Due to the uncommon use of tampon among Thai women, most of them are unfamiliar with inserting the device into their vagina. From our previous study, there was a concern that some women might not use the self-sample device properly [28]. There have been no previous studies compared self-sampling with standard methods for HPV screening in Thailand. We conducted this study to evaluate the agreement between self-sampling vaginal and physician-collected cervical HPV testing in Thai women.

## Main text

### Study populations

The study protocol was approved by the Institutional Review Board of Chulabhorn Hospital (No. 10/2013). We recruited women aged 30–70 years who visited a colposcopy clinic at Chulabhorn Hospital, Bangkok, Thailand during March 1 to June 30, 2015. Women were eligible for inclusion if they were attending the colposcopy clinic, aged 30–70 years, had no history of cervical cancer, had not undergone a hysterectomy, and were currently not pregnant.

### Sample collection

After the participants had given written informed consent to participate in the study, they received instructions by video made by research project’s staffs to explain how to use the vaginal self-sampling brush, verbal and illustrations for vaginal self-sampling. The vaginal specimens were first obtained by self-sampling with the Evalyn Brush (Rovers Medical Devices B.V., Oss, The Netherlands), which is a dry brush. Then, the participants were examined by a gynecological oncologist who obtained an endocervical sample with a Cervex-Brush (Rovers Medical Devices).

### Specimen preparation

The self- and physician-collected specimens were both suspended in 10 ml transport medium, SurePath Preservative Fluid (Becton, Dickinson and Company, USA).

### High-risk (hr)HPV testing

All self-sampled and physician-collected specimens were sent to the central laboratory of Chulabhorn Hospital for hrHPV testing. All samples were analyzed by Cobas4800 HPV test (Roche Molecular Diagnostics, Pleasanton, CA, USA) within 1 week after collection.

### Statistical analysis

Frequency, percentage, mean and standard deviation (SD) were used to calculate the general characteristics. The agreement levels were analyzed by Cohen’s kappa statistics. The statistical significance level was at 0.05. All data were analyzed by STATA/SE version 12.1.

### Results

We enrolled 250 eligible women. Two participants were excluded because of a history of cervical cancer and previous hysterectomy. One pair of samples was excluded because of invalid test results. The mean and median ages of the remaining 247 participants were 47.2 and 47.0 years (range 30–70 years; SD 9.8 years), respectively. Table 1 shows the baseline characteristics of the participants. Most participants were Thai (96.8%) and Buddhist (99.2%). Average age at first sexual intercourse was 22.5 years (range 14–47 years; SD 4.9 years).

**Table 1:** Baseline characteristics of 247 study participants

Characteristics	*n* (%)
Age (years)^a^	
30–39	61 (24.7)
40–49	87 (35.2)
50–59	68 (27.5)
60–70	31 (12.6)
Race	
Thai	239 (96.8)
Chinese	8 (3.2)
Religion	
Buddhist	245 (99.2)
Christian	1 (0.4)
Other	1 (0.4)
Education level	
Less than high school	69 (27.9)
High school	66 (26.7)
Bachelor degree	83 (33.6)
Higher than bachelor degree	29 (11.7)

^a^Mean age 47.2 years, standard deviation 9.8 years; median age 47.0 years

Overall hrHPV prevalence was 41.3% (102/247) from self-collected specimens and 36.0% (89/247) from physician-collected specimens. The prevalence of hrHPV 16, 18 and non-16, 18 from self- and physician-collected specimens is shown in Table 2.

**Table 2:** Prevalence of hrHPV from self- and physician-collected specimens

HPV	Prevalence	
	Physician-collected (%)	Self-collected (%)
hrHPV positive	36.0	41.3
hrHPV non-16/18	27.5	32.0
hrHPV 16	6.9	7.3
hrHPV 18	2.8	3.6

*hrHPV* high-risk human papillomavirus

The concordance of hrHPV test results between self- and physician-collected specimens is shown in Table 3. The concordance was 74.5% with moderate agreement and κ 0.46 for overall hrHPV. For hrHPV 16, the concordance was 96.4% with substantial agreement and κ 0.72. For hrHPV 18, there was 96.8% concordance with moderate agreement and κ 0.48.

**Table 3:** Concordance between hrHPV detection by self- and physician-collected method

Self-collected	Physician-collected		Agreement (%)	κ	Strength of agreement^a^	*P*
	Positive	Negative				
hrHPV						
Positive	64	38	74.49	0.46	Moderate	< 0.001
Negative	25	120				
hrHPV non-16, 18						
Positive	45	34	76.92	0.44	Moderate	< 0.001
Negative	23	145				
hrHPV 16						
Positive	13	5	96.36	0.72	Good	< 0.001
Negative	4	225				
hrHPV 18						
Positive	4	5	96.76	0.48	Moderate	< 0.001
Negative	3	235				

*hrHPV* high-risk human papillomavirus

^a^See Ref. [37]

### Discussion

We found that the prevalence of hrHPV from self-collected specimens (41.3%) was higher than from physician-collected specimens (36.0%). To explain, the self-collected specimens are a combination of cervical and vaginal cells. Additionally, the sampling order first obtained from the self-collected specimens may then collect higher number of exfoliated cells. In particular, the higher prevalence of low-risk HPV in the lower vagina than in the cervix and scanty cross-reactivity of the hrHPV assay to low-risk genotypes can partially explain this finding [18, 29].

Our study revealed 74.5% concordance between self- and physician-collected specimens in detecting overall hrHPV with κ 0.46, which showed moderate agreement. The level of agreement in our study was not as high as that in most previous studies [19–25]. Most previous studies found 70.6–94.2% concordance with κ 0.6–0.9, which represented substantial agreement between these two methods. However, some studies revealed the same level of agreement as in our study [29–31].

One study showed that the agreement between these two methods was lower among older women, which supports our results [30]. The median age of our participants was 47.0 years, which was higher than that in other studies (26.4–41.0 years) [19, 21–25], and ~ 40% of the participants were older than 50 years. This might be the reason why our study showed lower concordance and agreement than the other studies showed. Additionally, some women in this study reported that they were not confident about using the device correctly [28].

Although the agreement level of overall hrHPV between self- and physician-collected samples was moderate, the agreement level of HPV 16 was substantial, with concordance of 93.36% and κ 0.72. This finding for HPV 16 was the same as in the other studies [21, 25]. As mentioned above, the prevalence of HPV from self-collected specimens was higher than from physician-collected specimens. It might be then as a consequence that the concordance levels of other hrHPV were moderate. Whereas, HPV 16 was described in one previous study as the most prevalent HPV type in the cervical specimens, and especially with higher prevalence than in vaginal specimens [32]. Hence, these findings can partially explain about the high agreement and concordance levels of HPV 16.

### Limitations

All the participants in our study did the self-collection first then underwent pelvic examination to obtain physician-collected specimens later. This sampling order may have resulted in the self-collected specimens having more exfoliated cells than the physician-collected specimens had.

Our participants were women who attended a colposcopy clinic for various reasons such as abnormal cytology or positive HPV testing, so the prevalence of HPV in this group was higher than in the normal population. The prevalence of hrHPV in our study was 41.3 and 36.0% from self- and physician-collected specimens, respectively. The prevalence of hrHPV in Thai women in previous studies was 3.3–14.0% [33–36].

Due to the level of agreement in our study was slightly lower than in most previous studies, more studies with larger populations are needed to explore the reliability and feasibility of self-sampling of HPV as a method for cervical cancer screening in Thai and other Asian women. The molecular and biomarker analyses may be combined to achieve greater accuracy of the test.

## Abbreviations

HPV: human papillomavirus
hrHPV: high-risk human papillomavirus
